# A modified method for constructing experimental rat periodontitis model

**DOI:** 10.3389/fbioe.2022.1098015

**Published:** 2023-01-11

**Authors:** Xuyang Zhang, Minglu Xu, Qin Xue, Yao He

**Affiliations:** ^1^ Department of Orthodontics, Stomatological Hospital of Chongqing Medical University, Chongqing, China; ^2^ Chongqing Key Laboratory of Oral Disease and Biomedical Sciences, Stomatological Hospital of Chongqing Medical University, Chongqing, China; ^3^ Chongqing Municipal Key Laboratory of Oral Biomedical Engineering of Higher Education, Stomatological Hospital of Chongqing Medical University, Chongqing, China

**Keywords:** periodontitis, rat model, bone loss, animal model, inflammation

## Abstract

**Background:** Periodontitis is a prevalent disease caused teeth lost. The present rat models inducing periodontitis with thread ligature and metal steel ligature have some disadvantages.

**Methods:** We modified the existing rat ligature periodontitis model by fixing the thread ligature on the metal steel ligature passed through the gap between the first and second molars of rats with detailed modeling steps and illustrations. We research the pathological process of the periodontitis induced by the modified model, and briefly compared the modified model with the thread ligature model and the metal steel ligature model.

**Result:** Our experimental results showed that there was an aggravation in inflammatory infiltration and alveolar bone resorption in modeling area within 14 days of initial induction. After that, the inflammatory infiltration was reduced. And no significant increase in alveolar bone destruction appeared. The modified model was more reliable compared to the thread ligature model, and had greater ability of bacterial aggregation compared to the metal steel ligature model.

**Conclusion:** The modified method covered pathological process of the periodontitis, and showed sufficient efficiency and reliability in inducing rat periodontitis.

## Introduction

Periodontitis is a chronic inflammatory disease mainly caused by bacteria, and can cause periodontal tissue inflammation, alveolar bone recession and teeth lost, which seriously affects individual quality of life (Dannewitz et al., 2021; Kwon et al., 2021). Periodontitis is a prevalent disease worldwide (Nazir et al., 2020). It is estimated that 42% of American adults over the age of 30 suffer from periodontitis, among them 7.8% have severe periodontitis. Besides causing localized periodontal tissue damage (Eke et al., 2018), periodontitis is also closely associated with systemic diseases such as diabetes, atherosclerosis, rheumatoid arthritis and so on (Hajishengallis, 2015; Blasco-Baque et al., 2017; Chen et al., 2022).

Experimental animal models play a pivotal role in the study of disease development, histopathological changes, and therapeutic approaches (Robinson et al., 2019). Compared to cellular experiments, which are usually with a limited number of cell species, animal models have significant advantages. Since they are able to mimic the complex cellular environment of the human body. In addition, compared to human experiments, animal experiments allow for a good exploration of cause-and-effect relationships through medicine application and genetic modification. Such gain or loss of function studies are usually impossible in human clinical experimental studies due to ethical issues (Graves et al., 2008; Graves et al., 2012). Animal models of periodontitis are widely used in researches on the etiology exploration, treatment prevention, and tissue restoration.

Current animal models used to study periodontitis are usually established in rats, mice, dogs, pigs, and non-human primates (Ebersole et al., 2002; Liu et al., 2012; Liu et al., 2020; Lin et al., 2021). Compared with large animals, the choice of rats for the construction of periodontitis models has numerous advantages. First of all, in comparison with large animals such as dogs and pigs, rats have the characteristics of small size, low cost, strong environmental adaptability and high reproductive ability (Oz and Puleo, 2011; Wong et al., 2018), which greatly reduces the experimental cost and makes it possible to carry out experiments with a relatively large number of experimental animals. Secondly, gene editing engineering in rats or mice is much easier than in large animals, which is more conducive to exploring disease or drug mechanisms at the molecular level (Graves et al., 2008; Felder et al., 2017). Last but not least, availability of high-quality immunochemical and cellular reagents is also easier achieved in rat (Hajishengallis et al., 2011). Compared with mice, contribute to their larger oral space, rats may have more advantages in research about material in local alveolar bone reconstruction caused by periodontitis (Chen et al., 2021).

The placement of the thread ligature around the molar of experimental animals is by far the most common way to induce periodontitis. Placing the thread ligature around the teeth can induce rapid bacterial accumulation around periodontal tissues, which in turn leads to periodontitis symptoms such as loss of periodontal attachment tissue and alveolar bone resorption (Graves et al., 2012; Marchesan et al., 2018). However, due to the narrow oral space of rats and mice, there are some technical difficulties in constructing periodontitis models by this method, and improper operation during the modeling process may also cause traumatic injuries (Abe and Hajishengallis, 2013; Pereira et al., 2019). In addition, the thread ligature that induce bacteria colonization may be dislodged during the activities of experimental animals chewing and swallowing. And compared with mice, this phenomenon might more likely occur in rats, due to their powerful chewing activity. Metal steel ligature has also been reported for constructing animal periodontitis models in dogs, rats and mice because of their ability to promote local bacterial aggregation as well (Tubb et al., 1990; Liu et al., 2012; Li et al., 2020). The thread ligature induced and metal steel ligature induced periodontitis models in rats are two widely used periodontitis models at present. However, both models have disadvantages, such as the thread ligature is easy to lose, while, the ability of metal ligature to induce bacterial aggregation may be inferior to threads, due to its smooth surface.

Here, we modified this model by fixing the thread ligature on the metal steel ligature passed through the gap between the first and second molars of rats with detailed modeling steps and illustrations. Then, we observed the pathological process of the periodontitis induced by the modified model. And we briefly compared it with the thread ligature model and the metal steel ligature model in aspects of the degree of periodontal defects (alveolar bone resorption, osteoclast and inflammatory cell infiltration), reliability, and bacterial aggregation ability. This modified model is expected to provide some aids in the researches on periodontitis etiology exploration, treatment prevention, and tissue restoration.

## Materials and methods

### Animals

All animal experiments were performed in accordance with the Declaration of Helsinki, the National Institutes of Health Guide for Care and Use of Laboratory Animals, and were approved by the Animal Ethics Committee at Chongqing Medical University (AECCMU-2020-004).

Adult male Sprague–Dawley rats weighing 350–400 g at 10–12 weeks were purchased from the Animal Experimental Center of Chongqing Medical University. All the animals were housed in the Experimental Animal Center of Chongqing Medical University Stomatology Hospital and maintained in a specific-pathogen-free (SPF) environment at 25°C, 40% humidity, and a 12-h light/dark cycle, and the animals were free to get food and water.

Twenty eight rats were randomly divided into seven groups: the control group (CON), the 3 days group (3D), the 7 days group (7D), the 14 days (14D) group, the 21 days (21D) group, the 21 days thread ligature group (21T), and the 21 days metal steel ligature group (21M) (4 rats in each group). The modified periodontitis models were constructed on rats in the 3D group, 7D group, 14D group, and 21D group (The specific modeling steps were shown in Table 1). The thread ligature and metal steel ligature were respectively used for the induction of periodontitis in the 21T group and the 21M group rats.

**TABLE 1:** Detailed procedure of the modified method to induce experimental periodontitis.

Step	Treatments (using the left side of the maxillary molars as an example)	
1	Place a rubber band to separate the first and second molars. 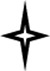 This step is in order to reduce the surgical trauma of the step 2 by pressing the gingival tissues between the first and second molars to the gingival side.	Hold the orthodontic elastic rubber band (5/16,2oz) with a needle holder in both hands; Pull the rubber band slightly to both sides to tighten the part at the holding site; Move the rubber band reciprocally along the gap between the first and second molars of the rat to stuck it in the gap between the first and second molars.
2	Insert the metal steel ligature.	Pull down the rubber band to tighten it; Insert the metal steel ligature (width = 0.2 mm) into the gap between the first and second molars of the rat, above the rubber band.
3	Attach the thread ligature to the metal steel ligature in the interdental space of the first/second molar.	Cut and pull out the rubber band used to separate the teeth; Tie the 3–0 thread ligature to the metal steel ligature on the buccal side; Slide the knot along the metal steel ligature to the buccal interdental space, and stick the thread on the palatal side of the knot into the interdental space of the first and second molars; Tie and fix the palatal thread ligature on the palatal metal steel ligature; Reserve a 1 mm long thread and cut off excess part;
4	Fix the metal steel ligature on the first molar.	Tighten the metal steel ligature to fixed it around the first molars of the rat; Reserve a 3 mm long metal steel ligature head and use gold crown shears to subtract excess part; Use resin to fix the steel ligature wire to the first molar.

### Establishment of experimental periodontitis in a rat model

**FIGURE 1:**
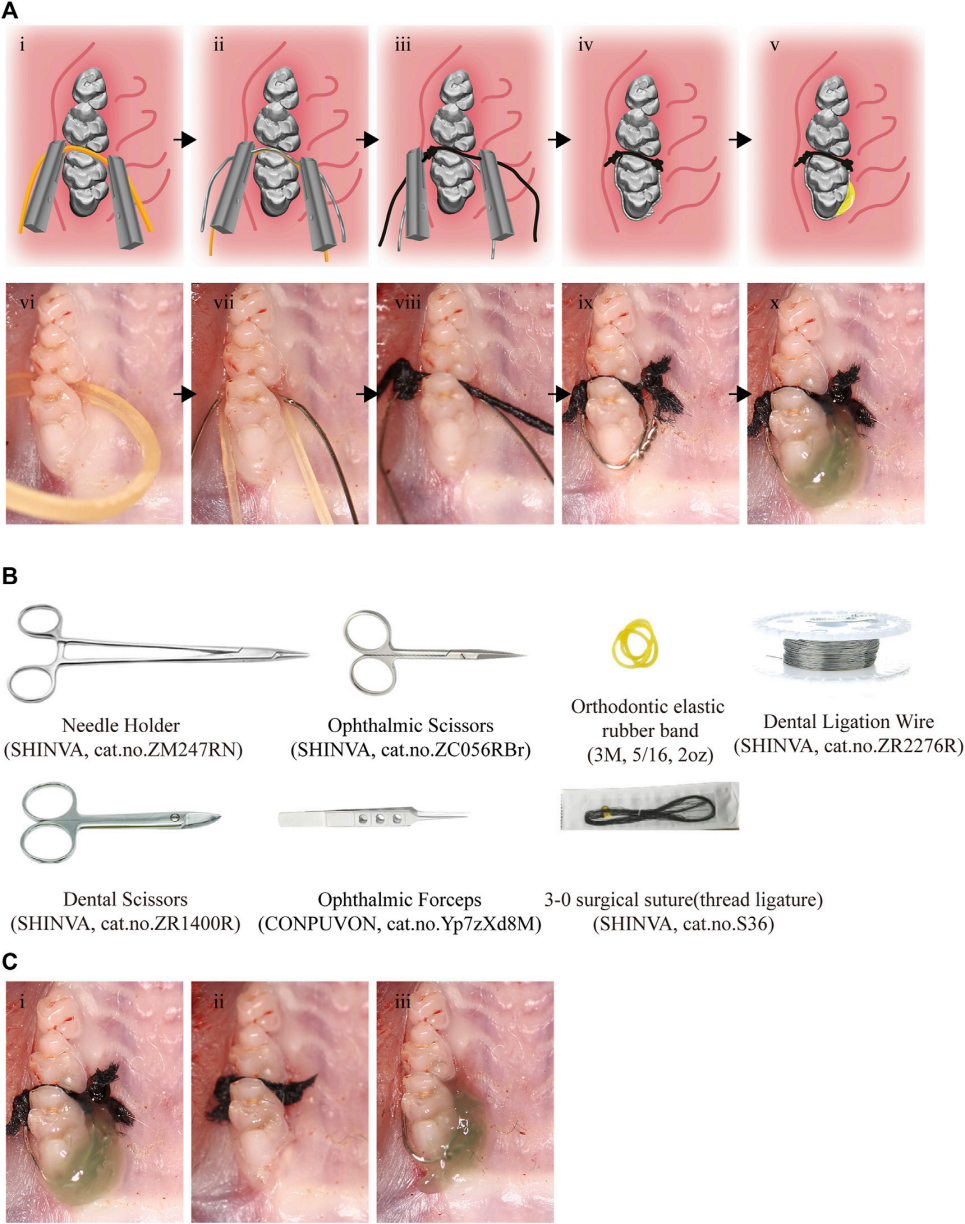
Procedures and tools for the effective and reliable experimental rat model. **(A)** i-v, The pattern diagram of the modeling procedures. vi-x, The photo of modeling procedures. Black arrows indicate the sequence of steps. **(B)** The tools for modeling. **(C)** The modeling completed photos of the modified model (i), thread ligature model (ii) and the metal steel model (iii).

Rats were anesthetized by an intraperitoneal injection of sodium pentobarbitone (35 mg/kg body weight) (Di Paola et al., 2011). In the modified periodontitis groups, periodontitis was induced by a combination of metal steel ligature (diameter = 0.2 mm) and thread ligature (3–0). Briefly, after separating the rats’ maxillary first molar and second molar with an orthodontic elastic rubber band (5/16, 2oz), a metal steel ligature with a section of knotted both ends thread ligature fixed on was placed between the first and second molars of the rat. And then the composite resin was used to fixed this model. The detail steps and illustrations were shown in Table 1 and Figure 1A. The equipment involved was listed in Figure 1B.

The CON group was subjected to the same operation but without placement of thread ligature and metal steel ligature. In the 21T group, a section of proximity 2 mm long thread ligature was inserted between the maxillary first and second molar of the rat, and both ends of the thread ligature were knotted to assist its stability. The metal steel ligature was placed around the maxillary first molar of the rat in the 21M group to induce periodontitis.

The modeling completed pictures of three methods can be seen in Figure 1C. All thread ligatures and metal steel ligatures were checked daily, and were replaced if they lost. The corresponding experimental groups of rats were euthanized by carbon dioxide asphyxiation at day 3, day 7, day 14, and day 21, respectively. After execution of the rats, maxillary alveolar bones were isolated and placed in 4% paraformaldehyde (PFA, Affymetrix, United States) overnight at 4°C for Micro-CT analysis, and histological staining. Thread ligatures and metal steel ligatures that induced periodontitis were collected for colony forming unit (CFU) count.

### Micro-CT analysis

The maxilla was scanned using a Scanco μCT40 scanner (Viva CT 40, Scanco Medical) at a resolution of 15 μm (70 KV,113 μA). The scan raw data were reconstructed to obtain a three dimensions reconstruction of the samples. To assess the degree of alveolar bone destruction in each sample, the distance from the enamel-cemental junction to the top of the alveolar crest (CEJ-ABC) between the first and second molars was measured. And the ratio of bone volume to tissue volume (BV/TV) of each sample were measured. The region of interest (ROI) was determined as an 18 × 18 pixel^2^ area on the alveolar bone between the first and the second molar, and the height is total 50 slices.

### Determination of bacterial accumulation

The collected thread ligatures and metal steel ligatures were gently washed with PBS to remove food residue and other debris. Subsequently, the thread ligatures and metal steel ligatures were placed in a 1.5 mL centrifuge tube containing 1 ml PBS and vortexed at 3,000 rpm for 2 min to extract the bacteria. Serial dilutions of the bacterial suspensions were plated on blood agar plates and CFU were counted after 7 days of anaerobic growth at 37°C (Abe and Hajishengallis, 2013).

### Paraffin section preparation

Followed CT scanning, the collected maxillary alveolars were decalcified with 19% ethylene diaminetetraacetic acid (EDTA; pH 7.5) at room temperature for 8 weeks. After decalcification, gradient alcohol was used for tissue dehydration and xylene was used for tissue transparency. Finally, after immersion in wax, the tissue was embedded in paraffin wax. When embedding, it is important to keep the buccal side of the tooth facing toward the bottom of the micro mold. Then sagittal sections (5 μm) were obtained for hematoxylin and eosin (H&E) staining, Tartrate‐Resistant Acid Phosphatase (TRAP) staining (Solarbio, China), and myeloperoxidase (MPO) staining.

### H&E staining

The slides were stained using the H&E staining kit (Solarbio, China) following the instructions provided by the manufacturer. Images of the modeling area with ×40 and ×200 magnification were obtained with microscope (BX41, Japan).

### TRAP staining

The TRAP stain was conducted with a Tartrate-Resistant Acid Phosphatase (TRAP) Stain Kit (Solarbio, China). The staining images were scanned and obtained with Olympus scanner (VS-200, Japan). The TRAP positive (+) cells, as osteoclast cells (clusters of more than three nuclei were counted as one osteoclast), in the alveolar bone between the maxillary first molar and the second molar were photographed and counted. The investigator was blinded to the groups while the data measurement and outcome evaluation were being performed.

### Myeloperoxidase (MPO) staining

Before staining, slides were baked at 65°C for 2 h. After deparaffinized and rehydrated, slides were subjected to antigen repair with 0.2% parenzyme (ZLI-9010, ZSGB-BIO, China) at 37°C for 15 min. Then the slides were stained according to the manufacturer’s instructions (SP-0022, Bioss, China). After PBS washed, slides were incubated with 0.3% hydrogen peroxide at 37°C for 20 min. After PBS washing, slides were incubated with goat serum at 37°C for 20 min to block reagent. Drain the slides and carefully wipe off the excess serum blocking reagent (do not rinse with PBS).

Slides were then incubated with anti-MPO primary antibody (Bioss, China, 1:100) at 4°C overnight. After PBS washed, the slides were incubated with goat anti-rabbit secondary antibody for 20 min at 37°C. After PBS washed, incubate samples with 1-3 drops of HSS-HRP for 20 min at 37°C. After PBS washed, the slides were then stained with 3,3-diaminobiphenyl tetrasodium (DAB) substrate for 20 s, and counterstained with hematoxylin. The staining images were scanned and obtained with Olympus scanner (VS-200, Japan). Count the MPO + cells (neutrophils, macrophages) in two random 100 × 100 μm^2^ square areas of the gingiva and periodontal ligament between the second molar and the first molar. The investigator was blinded to the groups while the data measurement and outcome evaluation were being performed.

### Statistical analyses

Differences between multiple groups were evaluated using one-way-ANOVA analysis (Tukey’s multiple comparisons test). Differences at *p* < 0.05 were considered significant. Statistical analyses were performed using the GraphPad Prism software version 8.0.2 (GraphPad Software, La Jolla, CA, United States).

## Results

### Modified periodontitis model accelerated the induction of alveolar bone destruction

**FIGURE 2:**
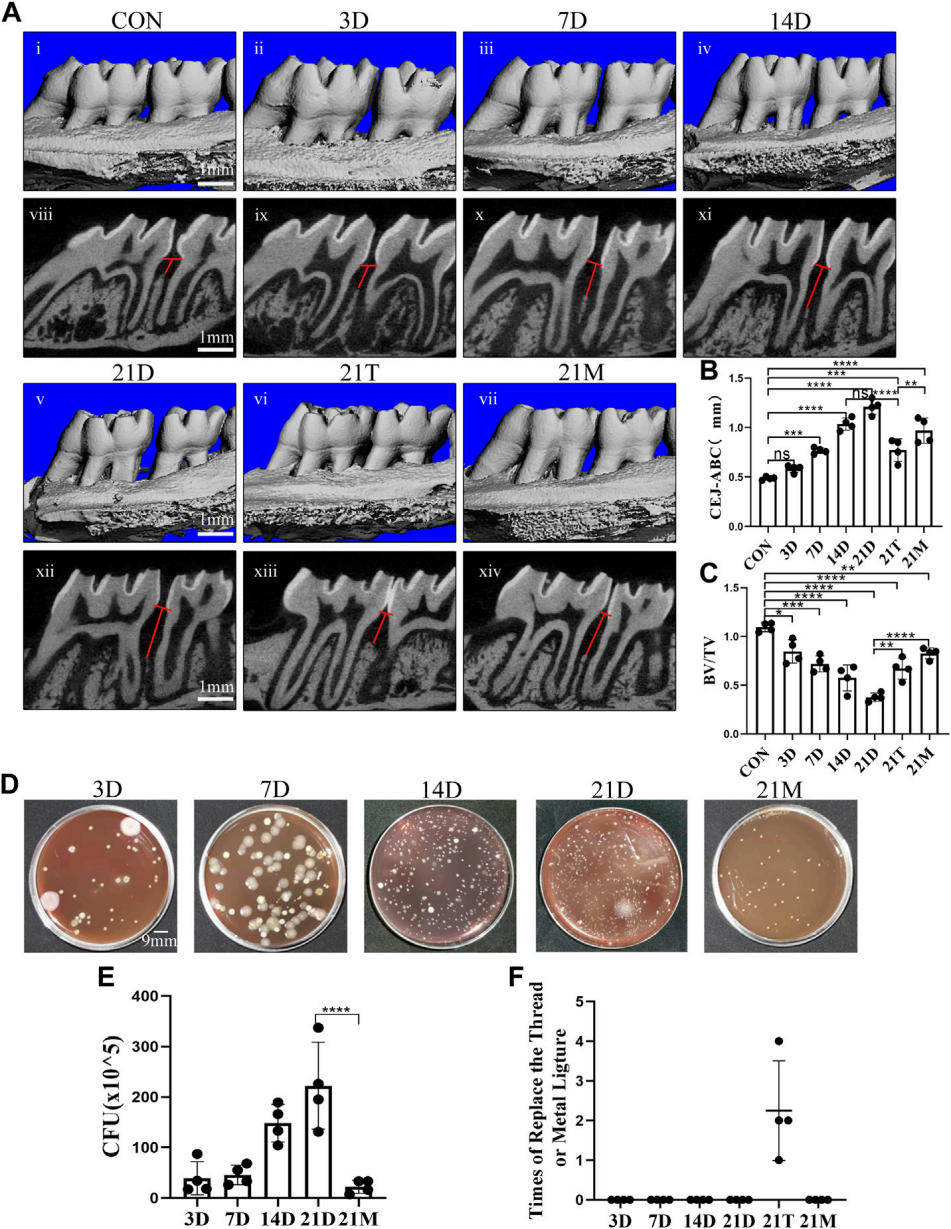
Degree of alveolar bone loss and bacteria accumulation after modeling. **(A)** Representative sagittal 3D (i-vii) and bi-dimensional views (viii-xiv) of the maxillary molars from CT scanning of the correspond groups. The red line corresponds to the distances from the cementoenamel junction to the alveolar bone crest (CEJ–ABC). **(B, C)** Measurement of the distance from the CEJ to the ABC and the ratio of bone volume to tissue volume (BV/TV) at each group after modeling. **(D)** Photograph of colony forming unit (CFU) from the 3D group, the 7D group, the 14D group, the 21D group and the 21M group. **(E)** Statistical analysis of colony forming unit (CFU) count. **(F)** Times of replace the thread ligature or metal steel ligature. Results are the mean ± s.d. (n = 4 rat per group). *p < 0.05, **p < 0.01, ***p < 0.001, ****p < 0.0001, ns no significance (one-way ANOVA and Bonferroni’s post hoc tests).

In order to observe the alveolar bone destruction of rats in modified periodontitis model, we performed Micro-CT analysis on the samples. It was observed that the degree of alveolar bone resorption showed an overall increasing trend during the induction periods of the modified periodontitis model. The distance of alveolar bone resorption did not increase significantly during the first 3 days of modeling, and began to stabilize after 14 days of modeling. Compared the 21D group with the 14D group, there was a no significant increase in the distance of alveolar bone resorption (Figures 2A–C).

It could be observed that after 21 days of induction, the alveolar bone resorption distance induced by the modified model was more obvious than those from the 21T group and the 21M group. In addition, after 21 days periodontitis induction, the degree of alveolar bone resorption induced by the thread ligatures was similar to the one induced by the modified model of the 7D group, and alveolar bone resorption degree induced by the metal steel ligatures was similar to the one induced by the modified model of the 14D group (Figures 2A–C).

### Modified periodontitis models promoted microbial aggregation

In the initiation and progression of periodontitis, dysbiotic microbial communities consisting predominantly of anaerobic bacteria, play an important role (Yu and Van Dyke, 2020). It is technically difficult to quantify microorganisms in rat periodontal tissues. However, in a rat periodontitis model, the number of microorganisms in periodontal tissues could be indirectly reflected by the ones on the thread ligatures and the metal steel ligatures that induce bacterial aggregation. Therefore, to reflect microbial aggravation in rat periodontal tissue, bacteria from thread ligatures and metal steel ligatures were extracted and anaerobically cultured for CFU counting. We observed that during the modified model inducing period, bacterial aggregation appeared a growing trend and peaked at the 21st day (Figures 2D, E).

Compared with the metal steel ligature group, more bacterial accumulation took place in the modified periodontitis model group (Figures 2D, E). There was no ligature or metal steel ligature lost in the 21D and 21M group, during the process of 21 days inducing. While the thread ligatures of the 21T group all lost for several times (Figure 2F). And although the lost thread ligatures were replaced promptly each time, this group was not included in the comparison because of the large fluctuations in the time of bacterial accumulation.

### Osteoclast infiltration in the modified periodontitis model

**FIGURE 3:**
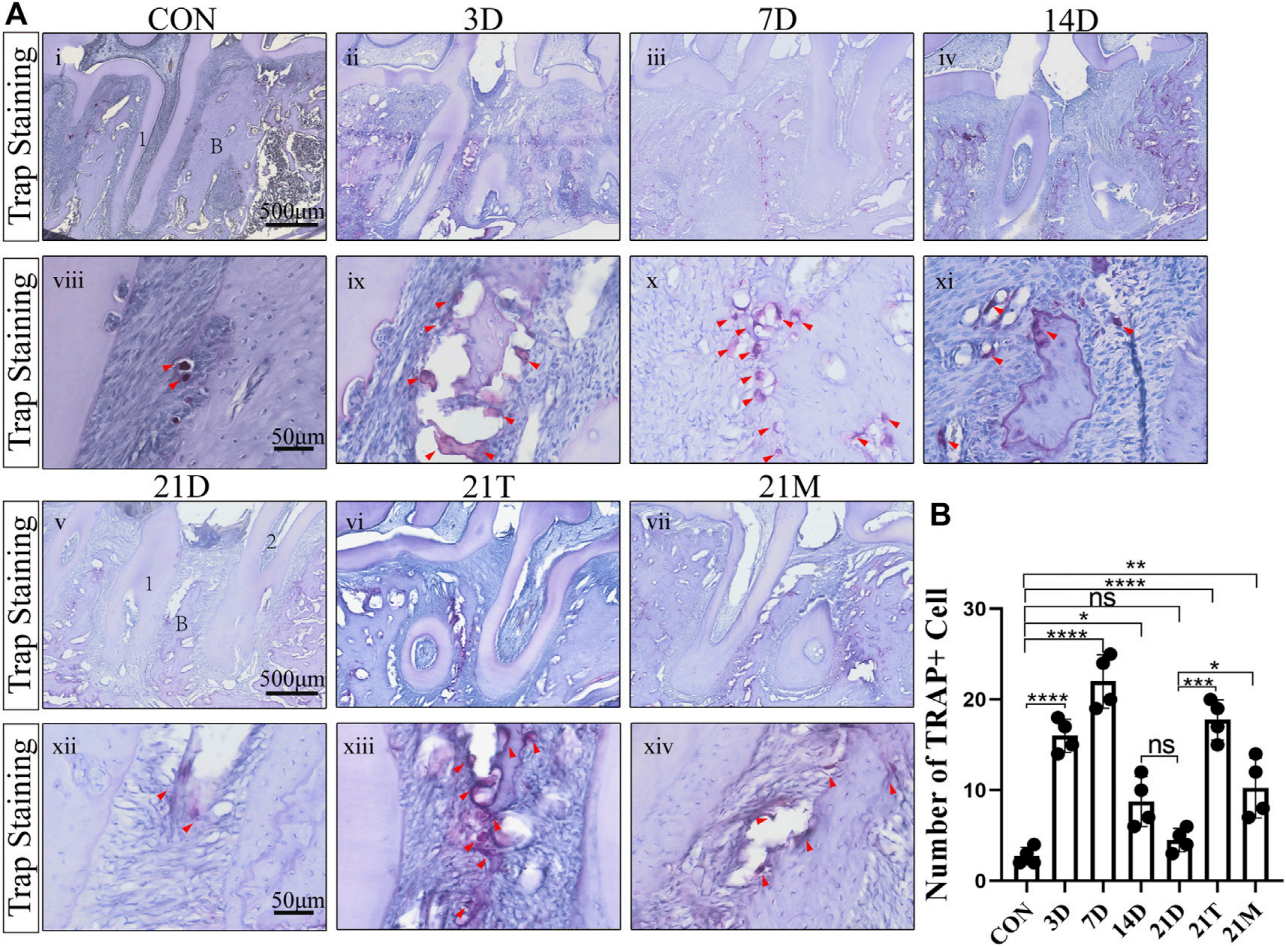
The degree of osteoclast infiltration **(A)** Representative TRAP-stained sections of periodontal tissues harvested from each group at low (i-vii) and high (viii-xiv) magnifications. 1, the root of the first molar; 2, the root of the second molar; B, alveolar bone. Arrowheads mark osteoclasts. **(B)** Total osteoclast number between the first and the second molars. Results are the mean ± s.d. (n = 4 rat per group). *p* < 0.05, **p < 0.01, ***p < 0.001, ****p < 0.0001, ns no significance (one-way ANOVA and Bonferroni’s *post hoc* tests).

To observe the osteoclastic activity in the periodontal tissue after periodontitis induction, we performed TRAP staining on the samples. The number of osteoclasts in the periodontal tissue elevated in all the modeled groups compared with the control group. A significant increase in osteoclasts occurred at the 3th days of induction by the modified model and reached a maximum at the 7th day. The number of osteoclasts decreased gradually after 7 days induction and stabilized on the 14th day. There was a no-significant decrease of the osteoclasts number between the 14D and 21D groups (Figure 3).

It was observed that the number of osteoclasts in the alveolar bone from the 21D group was less than those in the 21T group and the 21M groups. In addition, similar to the results of the degree of alveolar bone resorption, the osteoclast infiltration condition of the 21T group and the 21M group approximated with those of the 7D group and the 14D group, respectively (Figure 3).

### Inflammatory cell infiltration in the modified periodontal

**FIGURE 4:**
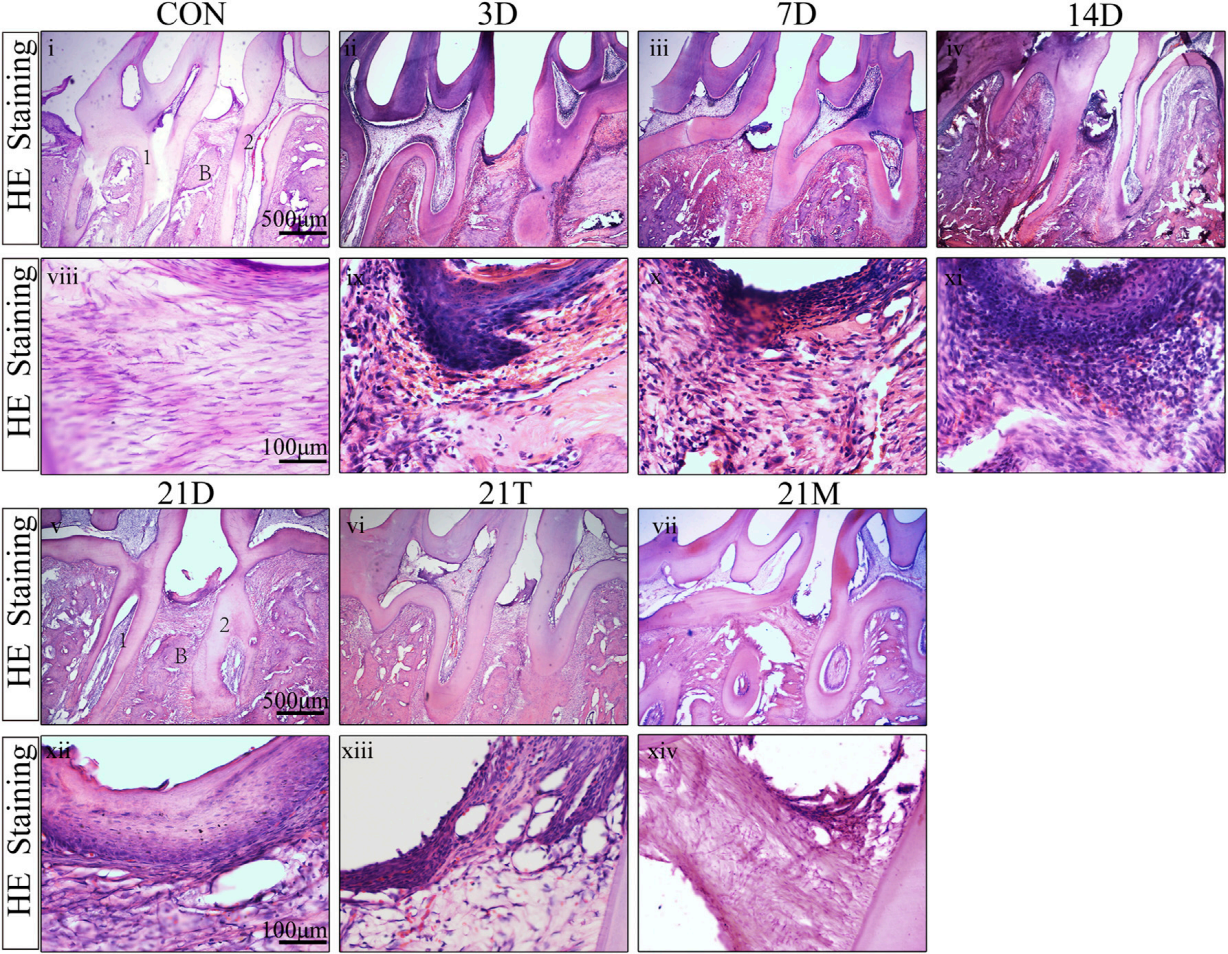
H&E staining result Representative H&E stained sections of periodontal tissues harvested from each group at low (i-vii) and high (viii-xiv) magnifications. 1, the root of the first molar; 2, the root of the second molar; B, alveolar bone.

**FIGURE 5:**
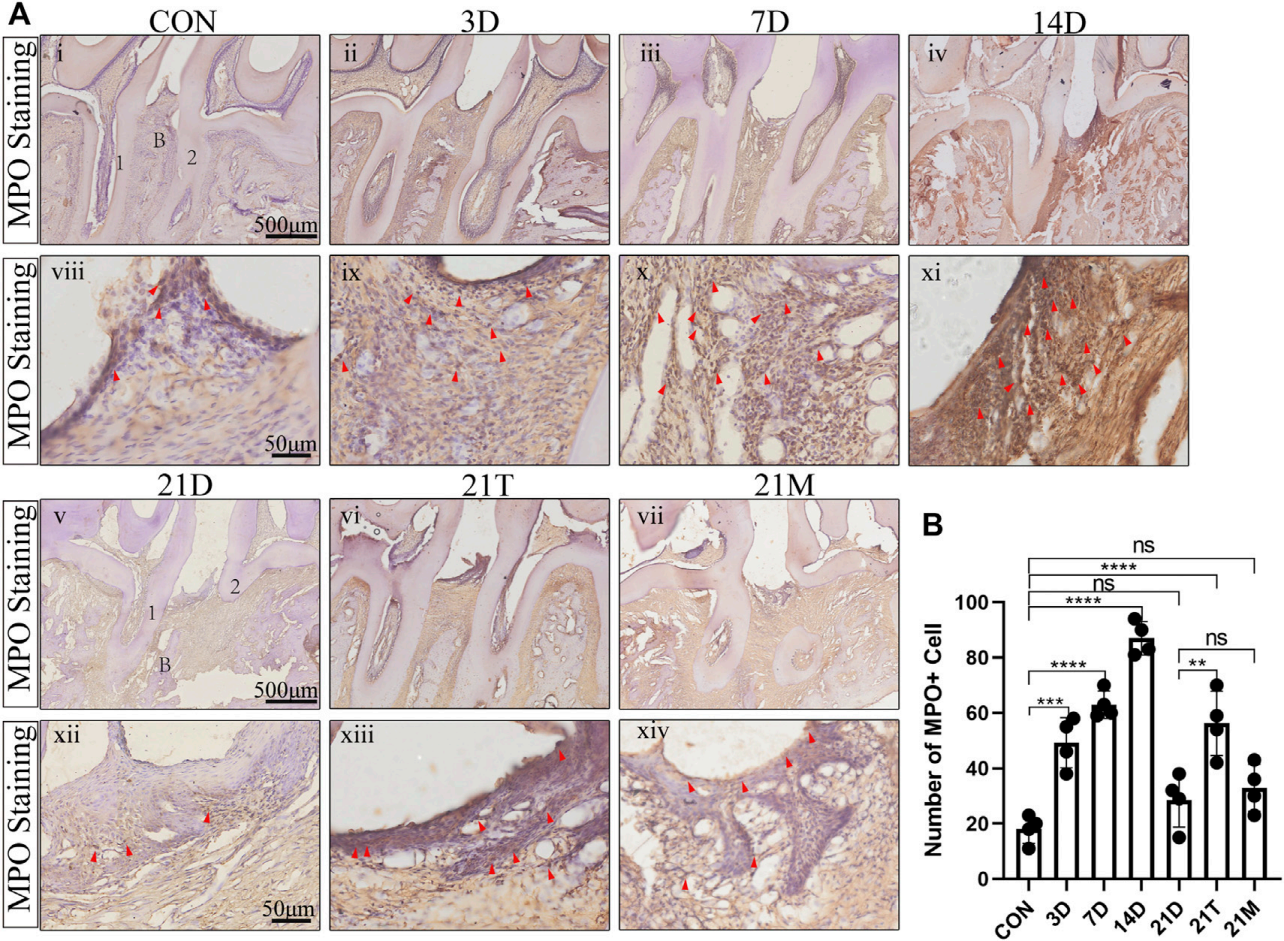
The degree of MPO + cells infiltration **(A)** Representative MPO + stained sections of periodontal tissues harvested from each group at low (i-vii) and high (viii-xiv) magnifications. 1, the root of the first molar; 2, the root of the second molar; B, alveolar bone. Arrowheads mark MPO + cell. **(B)** Total MPO + cell number from two random 100 × 100 μm^2^ square areas of the gingiva and periodontal ligament between the second molar and the first molar. Results are the mean ± s.d. (n = 4 rat per group). *p* < 0.05, **p < 0.01, ***p < 0.001, ****p < 0.0001, ns no significance (one-way ANOVA and Bonferroni’s *post hoc* tests).

To observe the histologic features of periodontitis, we performed the H&E staining in the slides. Besides the loss of connective tissue attachment and alveolar bone resorption, the infiltration of inflammatory cells could be seen in gingival tissues and periodontal ligaments between the distal root of the first molar and the mesial root of the second molar (Figure 4). To quantify the degree of inflammation, the MPO staining was performed, which MPO + cells represent neutrophils and macrophages. The results from MPO staining suggested significant inflammatory cell infiltration in periodontal tissues after modeling for day 3 to day 14 compared to the CON group. During 14 days of modeling, the number of MPO + cells showed an increasing trend, then declined to comparably level to the CON group at the 21 s t day (Figure 5). This suggested that although the alveolar bone resorption of the modified model group had been in a stable phase after 7 days induction of periodontitis (Figures 2A–C), the inflammatory state was still in an active phase until 14 days.

It was seen that after 21 days of induction, the degree of inflammatory cell infiltration in periodontal tissues was similar between the 21D group and the 21M group, and both had a lower number of MPO + cells than the 21T group, with MPO + cells approximated with those of the 7D group (Figure 5).

## Discussion

Periodontitis is a chronic inflammatory disease mainly caused by bacteria, and may result in periodontal tissue inflammation, alveolar bone recession, teeth lost and some systematic diseases (Hajishengallis, 2015; Blasco-Baque et al., 2017; Dannewitz et al., 2021; Kwon et al., 2021; Chen et al., 2022). Animal model is an important way to study the process and mechanism of disease, as well as related treatment methods (Robinson et al., 2019). Dogs, non-human primates, rats, mice and other different animals have been used to build periodontitis models (Ebersole et al., 2002; Liu et al., 2012; Liu et al., 2020; Lin et al., 2021). Compared with large animals, rats and mice have the advantages of rapid reproduction, low cost, easily feeding and genetic modification, thus being the widely used animal models for studying periodontitis (Graves et al., 2008; Hajishengallis et al., 2011; Oz and Puleo, 2011).

Current methods for constructing periodontitis include oral gavage of periodontitis-causing bacteria and stimulus induction around periodontal tissue that facilitates bacterial accumulation (Graves et al., 2008; Graves et al., 2012). Compared with the gavage model, the stimulus induced periodontitis model has the advantages of better control of the modeling area, shorter modeling time and so on (de Molon et al., 2016; Tamura et al., 2021). It has been proved that placing the thread ligature around the teeth could induce rapid bacterial accumulation around periodontal tissues, which in turn leaded to periodontitis symptoms such as loss of periodontal attachment and alveolar bone resorption (Graves et al., 2012; Marchesan et al., 2018). However, due to the smooth surface of teeth, the narrow space in rats’ and mice’s mouth, and the softness of the thread ligature, it is technically difficult to firmly fix the thread ligature on the teeth. Improper operation during the modeling process may also cause traumatic injuries (Abe and Hajishengallis, 2013; Pereira et al., 2019). Animals’ oral activities such as chewing and swallowing may also cause dislodgement of the thread ligature. Compared with placing a thread ligature around the molar, inserting a section of thread ligature knotted at both ends between the first and second molars of the rat, may cause more chances of dislodgement. And this phenomenon might more likely occur in rats, due to their powerful chewing activity.

The orthodontic metal steel ligature have also been reported to be used in the construction of periodontitis models (Tubb et al., 1990; Liu et al., 2012). Compared with thread ligatures, metal steel ligatures have more robust strength, which can be more easily and firmly fixed on rat molars. Li et al. used orthodontic metal steel ligatures to simplify the construction of a periodontitis model in mice (Li et al., 2020). However, due to the smooth surface of the metal steel ligature, its ability to induce bacterial aggregation may not be as well as that of the thread ligature. Therefore, we modify the existing model of periodontitis, in order to build a more reliable and effective model, and provide aids for future periodontitis related experiments.

By fixing the thread ligature on the metal steel ligature passed through the gap between the first and second molars of rats, we modified the traditional ligature inducing periodontitis model and provided the detail modeling steps. Preserving the advantage of thread ligature in bacteria aggregation, the usage of metal steel ligature reduces the difficulty and risk of tissue trauma during the insertion of thread ligature. In addition, in order to reduce the surgical trauma further, we used an orthodontic rubber band for tooth apart during the modeling process, which can protect the periodontal tissues by pressing the gingival tissues between the first and second molars to the gingival side.

The pathological process of the periodontitis has been covered sequentially with this modified model. The Micro-CT results showed that significant alveolar bone resorption occurred after inducing by the modified model for 7 days, and the level of bone resorption stabilized after inducing for 14 days. TRAP staining results showed that the number of osteoclasts increased significantly after 3 days of modeling and reached a peak on day 7, after which it was gradually decreased. H&E staining and MPO staining results suggested a significant inflammatory cell infiltration at day 3 after modeling, and the degree of inflammatory cell infiltration began to decrease after modeling for 14 days. De Molon et al. divided the process of ligature induced periodontitis in rats into two sequential processes: the acute process of bone destruction and the chronic phase (de Molon et al., 2018). They concluded that in the acute process of periodontitis, inflammatory cell infiltration was evident and alveolar bone resorption was rapid. Whereas in the chronic phase, the number of infiltrating inflammatory cells decreased and alveolar bone resorption slowed down (de Molon et al., 2018). The histological analysis results from mouse periodontitis model by Marchesan et al. (2018) also showed that periodontitis in mice went through the same two phases. Rat periodontitis induced by the modified method also experienced these similar two stages. In this experiment, combined with CT results and histological staining results, we believe that the pathological process of periodontitis kept in the active phase within 14 days of the modified model induction and stayed in the chronic phase from day 14 to day 21. Subsequent experiments using this modified model could refer to these time points.

It can be observed that after inducing for 21 days, rats in the 21D group had a more pronounced alveolar bone resorption compared to those from the 21T group and the 21M groups. The results showed that the 21T group had similar level of alveolar bone resorption, osteoclast and inflammatory cell infiltration to the 7D group. While situations of the 21M group was equivalent to the 14D or the 21D group. The results showed that modified model could bring more obvious periodontal defects compared with the two others. There was no ligature or metal steel ligature lost in the 21D and 21M group. While the thread ligatures of the 21T group all lost for several times. The results of the CFU experiment suggested that the modified model had more bacterial aggregation compared to the metal steel model, indicating that thread ligature largely elevated the number of bacteria aggregating around periodontal tissues after 21 days induction. The above experiments suggested that the modified model was more reliable compared to the thread ligature model, and it could better promote the aggregation of bacterial microorganisms than the metal steel ligature model.

It is worth noting that the main idea behind the design of this modified model is to combine the robustness of the metal steel ligature model with the excellent bacterial aggregation ability of the thread ligature model. The thread ligature fixed on the metal steel ligature can be either a section of knotted both ends thread ligature placed between the first and second molars of the rat, or a loop around the first and/or second molar (Supplementary Figure S1). In this study, we chose the way of knotted ends thread. The purpose was to design a model of periodontitis that induced localized periodontal tissue loss for providing primary model related to local alveolar bone reconstruction (Greenstein et al., 2009; Liu et al., 2019). CT screenshots from study inducing periodontitis using a section of both ends knotted thread ligature showed significant alveolar bone resorptions in the modeled area, while no significant changes appeared in the non-modeled area (Marchesan et al., 2018). However, in the present modified model, we observed alveolar bone resorptions occurred in both proximal and distal alveolar crests of the first molar. We speculate it may be attributed to that the circling metal steel ligature also caused bacterial accumulation in the proximal of the first molar. And the metal steel ligature may also have caused some mechanical irritation to the periodontium, which promoted periodontitis aggravation (Li et al., 2021). Based on this speculation, this modeling method might be modified *via* reducing the ligature wire encircling area and combining with composite resin bonding to enhance retention in the future.

In oral clinic, periodontitis is mainly an inflammatory disease caused by bacteria as an initiating factor. Under the stimulation of plaque, inflammatory cells infiltrate in local periodontal tissue and osteoclasts differentiate, resulting in the destruction of alveolar bone absorption (Yamaguchi et al., 2017; Usui et al., 2021). Similar to the traditional rat periodontitis model, this modified model also caused local periodontitis by simulating bacterial aggregation in periodontal tissue and cause alveolar bone resorption (Graves et al., 2012; Marchesan et al., 2018). What’s more, after 21 days induction, the modified model showed high stability, enough bacteria aggregation, and distinct alveolar destruction compared to traditional ones, which make this model feasible to be applied in the mechanism or treatment researches related to periodontitis.

However, there are also several limitations that should be considered. At first, although in order to protect the gums during the modeling process, the rubber bands are used to divide teeth, this step makes the modeling process more complicated. Secondly, it seems difficult to construct this modified model in mice, due to the narrower oral space. Thirdly, since this experiment only include the results of traditional models after 21 days of induction, it could not reflect the pathological process of periodontitis in their specific situation. The periodontal tissue performance should be detected in different continuous sequential time durations for more details. Last but not least, in this experiment, we do not explore whether the bacteria induced by ligatures is same with that in human. The specific bacteria induced periodontitis in rats might be different with that in human (Marchesan et al., 2018). Further exploration on bacteria composition and pathogenic mechanism would make this modified model more convincing.

In conclusion, we provided a modified method for the induction of periodontitis in rats and gave a detailed modeling procedure. The results showed that this model covered the sequential pathological process of the periodontitis from the acute stage to the chronical stage with sufficient reliability and efficiency in inducing periodontitis. This modified model is expected to provide some aids in the research about the mechanism of periodontitis and the effect of different treatments methods or material.

## Data Availability

The original contributions presented in the study are included in the article/Supplementary Material, further inquiries can be directed to the corresponding author.
