# Corrigendum: A modified method for constructing experimental rat periodontitis model

**DOI:** 10.3389/fbioe.2023.1177628

**Published:** 2023-03-29

**Authors:** Xuyang Zhang, Minglu Xu, Qin Xue, Yao He

**Affiliations:** ^1^ Department of Orthodontics, Stomatological Hospital of Chongqing Medical University, Chongqing, China; ^2^ Chongqing Key Laboratory of Oral Disease and Biomedical Sciences, Stomatological Hospital of Chongqing Medical University, Chongqing, China; ^3^ Chongqing Municipal Key Laboratory of Oral Biomedical Engineering of Higher Education, Stomatological Hospital of Chongqing Medical University, Chongqing, China

**Keywords:** periodontitis, rat model, bone loss, animal model, inflammation

In the published article, there was an error in Figure 2 as published. In the Figure 2E, the “21T” should be revised to the “21M”. The corrected Figure 2 and its caption “Degree of alveolar bone loss and bacteria accumulation after modeling” appear below.

**FIGURE 2:**
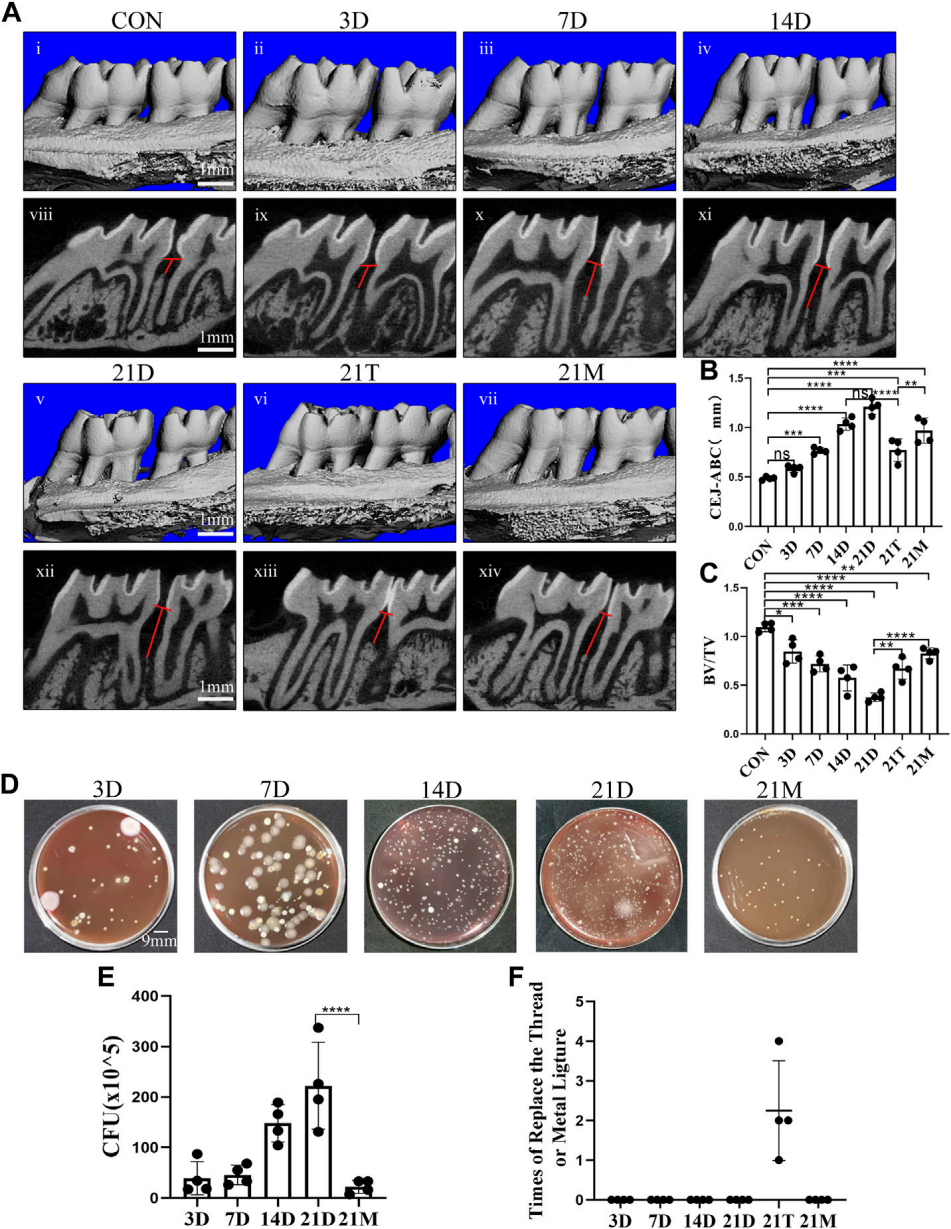
Degree of alveolar bone loss and bacteria accumulation after modeling. **(A)** Representative sagittal 3D (i-vii) and bi-dimensional views (viii-xiv) of the maxillary molars from CT scanning of the correspond groups. The red line corresponds to the distances from the cementoenamel junction to the alveolar bone crest (CEJ–ABC). **(B, C)** Measurement of the distance from the CEJ to the ABC and the ratio of bone volume to tissue volume (BV/TV) at each group after modeling. **(D)** Photograph of colony forming unit (CFU) from the 3D group, the 7D group, the 14D group, the 21D group and the 21M group. **(E)** Statistical analysis of colony forming unit (CFU) count. **(F)** Times of replace the thread ligature or metal steel ligature. Results are the mean ± s.d. (n = 4 rat per group). *p < 0.05, **p < 0.01, ***p < 0.001, ****p < 0.0001, ns no significance (one-way ANOVA and Bonferroni’s *post hoc* tests).

The authors apologize for this error and state that this does not change the scientific conclusions of the article in any way. The original article has been updated.

