# Supplementary material for: A modified method for constructing experimental rat periodontitis model
Source: Front Bioeng Biotechnol. 2023 Jan 11;10:1098015. doi: 10.3389/fbioe.2022.1098015 (PMC9873956; doi:10.3389/fbioe.2022.1098015)
Supplement: Supplementary file 1 [file Image1.pdf]

Supplementary Material

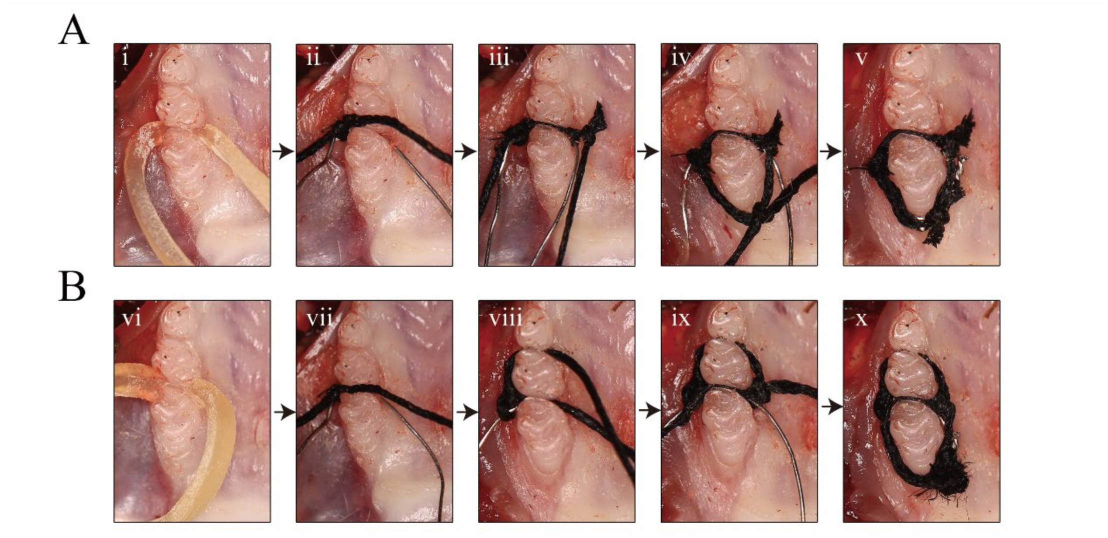

Figure S1 : Procedures of modified models with thread around molars.

(A) Procedures of the modified model by a loop thread ligature fixed on the metal steel ligature around the first molars of the rat. **(B)** Procedures of the modified model by a loop thread ligature fixed on the metal steel ligature around the first and the second molars of the rat.
